# Rac1 in podocytes promotes glomerular repair and limits the formation of sclerosis

**DOI:** 10.1038/s41598-018-23278-6

**Published:** 2018-03-22

**Authors:** Rin Asao, Takuto Seki, Miyuki Takagi, Hiroyuki Yamada, Fumiko Kodama, Yoshiko Hosoe-Nagai, Eriko Tanaka, Juan Alejandro Oliva Trejo, Kanae Yamamoto-Nonaka, Yu Sasaki, Teruo Hidaka, Takashi Ueno, Motoko Yanagita, Yusuke Suzuki, Yasuhiko Tomino, Katsuhiko Asanuma

**Affiliations:** 1 Medical Innovation Research, TMK Project, Kyoto University Graduate School of Medicine, Kyoto, Japan; 2 Division of Nephrology, Juntendo University Faculty of Medicine, Tokyo, Japan; 3 Department of Nephrology, Kyoto University Graduate School of Medicine, Kyoto, Japan; 4 Department of Pediatrics, Tokyo Medical and Dental University, Tokyo, Japan; 5 Laboratory of Proteomics and Biomolecular Science, Research Support Center, Juntendo University Faculty of Medicine, Tokyo, Japan; 6 Department of Nephrology, Chiba University Graduate School of Medicine, Chiba, Japan

## Abstract

Rac1, a Rho family member, is ubiquitously expressed and participates in various biological processes. Rac1 expression is induced early in podocyte injury, but its role in repair is unclear. To investigate the role of Rac1 expression in podocytes under pathological conditions, we used podocyte-specific Rac1 conditional knock-out (cKO) mice administered adriamycin (ADR), which causes nephrosis and glomerulosclerosis. Larger areas of detached podocytes, more adhesion of the GBM to Bowman’s capsule, and a higher ratio of sclerotic glomeruli were observed in Rac1 cKO mice than in control mice, whereas no differences were observed in glomerular podocyte numbers in both groups after ADR treatment. The mammalian target of rapamycin (mTOR) pathway, which regulates the cell size, was more strongly suppressed in the podocytes of Rac1 cKO mice than in those of control mice under pathological conditions. In accordance with this result, the volumes of podocytes in Rac1 cKO mice were significantly reduced compared with those of control mice. Experiments using *in vitro* ADR-administered Rac1 knockdown podocytes also supported that a reduction in Rac1 suppressed mTOR activity in injured podocytes. Taken together, these data indicate that Rac1-associated mTOR activation in podocytes plays an important role in preventing the kidneys from developing glomerulosclerosis.

## Introduction

Glomerulosclerosis is present in a majority of kidney diseases that progress to a chronic state. Several glomerular diseases dramatically accelerate podocyte injury leading to a loss of podocytes from the glomerular basement membrane (GBM). Studies on nephrosis in human type I and II diabetic nephropathy, in IgA nephropathy, and in several rat and mouse models collectively provide strong evidence of a correlation between the loss of podocytes and the progression of glomerular diseases. Selective and irreversible injury to podocytes caused glomerulosclerosis in well-designed transgenic mice and rats^1,2^. Without compensation, progressive glomerular damage develops further with podocyte loss, and the formation of synechiae by the attachment of parietal epithelial cells to the denuded GBM occurs as the initial step to glomerulosclerosis^3,4^. To date, no clear evidence has been found for the direct replacement of lost podocytes via replication of the remaining podocytes.

Rho family GTPases regulate actin reorganization^5^. The Rho family of small GTPases consists of 3 major members, RhoA, Rac1, and Cdc42, which control signal-transduction pathways that influence various aspects of cell behaviour, including cytoskeletal dynamics^6–8^, cell adhesion^5^, cell growth^9^, and super oxide formation^10,11^. Several studies have described the function of small GTPases in the kidneys. Synaptopodin is an actin-associated protein that regulates RhoA and Cdc42 signaling and cell migration in podocytes^12,13^. Cdc42 is necessary for the maintenance of podocyte structure and function^14^. Scott *et al*. demonstrated that podocyte-specific inactivation of Cdc42, but not Rac1 or RhoA, led to congenital nephropathy^15^.

The Rac subclass has 3 members: Rac1, Rac2, and Rac3^16^. Rac1 is ubiquitously expressed and has various functions, while Rac2 expression is restricted to hematopoietic lineage cells. Rac3, the most recently described isoform, is expressed in the brain, lungs, liver, and pancreas^16^. The role of Rac1 in podocytes under pathological conditions is unresolved; however, some studies on the role of Rac1 in podocytes have been recently published. Shibata *et al*. found that Rac1 serves as a key regulator of non-aldosterone-mediated mineralocorticoid receptor activation. They identified a cross-talk pathway between a mineralocorticoid receptor, which is a nuclear transcription factor regulated by the steroid aldosterone, and Rac1, which has been implicated in proteinuric kidney disease^17^. Ishizaka *et al*. reported that podocyte-specific Rac1 deletion resulted in morphological alterations to podocytes and a lower number of podocytes remaining during the progression of diabetic nephropathy^18^. Following growth factor stimulation, Rac1 functions as a critical regulator of expression of both mammalian target of rapamycin complex 1 (mTORC1) and mTORC2, which in turn control cellular size^19^. As podocytes are terminally differentiated epithelial cells, the only way to immediately compensate for lost podocytes involves cellular hypertrophy and migration to cover the denuded GBM with remaining podocytes. A number of studies have reported previously in which podocyte hypertrophy was observed in various disease models^20–22^.

Here, we demonstrated that Rac1 deletion induced podocyte loss and glomerulosclerosis in mice with adriamycin (ADR)-induced nephropathy, and we addressed the role of Rac1 in podocytes during the process of glomerulosclerosis development.

## Materials and Methods

### Mouse strains

This study was approved by Institutional Review Board of Juntendo University and Kyoto University and was conducted according to the Declaration of Helsinki. The mouse strain with floxed Rac1 alleles used in the study was described elsewhere^23^. A podocyte-restricted Rac1 knockout mutant was generated by crossing floxed mice with Nphs2-Cre transgenic mice^24^. All mice had a BALB/c background.

### Antibodies

A polyclonal rabbit anti-Rac1 antibody (Abcam, Cambridge, UK), a monoclonal mouse anti-Rac1 antibody (Merck Millipore, Frankfurt, Germany), a polyclonal rabbit anti-Rac1/2/3 antibody (Cell Signaling Technology (CST), Massachusetts, USA), a polyclonal rabbit anti-WT-1 antibody (Santa-Cruz Biotechnology, Dallas, USA), a monoclonal mouse anti-synaptopodin antibody (Progen, Heidelberg, Germany), a monoclonal mouse anti-GAPDH antibody (Abcam, Cambridge, UK), a monoclonal mouse anti-β-actin antibody (Sigma-Aldrich, St Louis, CO, USA), a monoclonal mouse anti-pS6 antibody (CST), a monoclonal mouse anti-S6 antibody (CST), a polyclonal rabbit anti-pPAK1 antibody (CST), and a polyclonal rabbit anti-PAK1 antibody (CST) were purchased for immunohistochemistry or western blot (WB) analysis.

### Podocyte culture and Rac1 knockdown (KD) by short hairpin RNAs (shRNAs)

Conditionally immortalised mouse podocytes were cultured as described previously^25^. To generate Rac1 KD podocytes, we used a Gateway System (BLOCK-iT U6 RNAi Entry Vector Kit and BLOCK-iT Adenoviral RNAi Expression System; Invitrogen, CA, USA) following the manufacturer’s instructions. Briefly, adenoviral particles expressing Rac1 shRNA were produced in 293 A cells. LacZ non-silencing adenoviral shRNA was used as control. Differentiated cultured podocytes were transduced with the adenoviral particles and transduction was confirmed by sodium dodecyl sulphate-polyacrylamide gel electrophoresis (SDS-PAGE) and WB analysis. For the ADR-administration experiments, control and Rac1 KD podocytes were cultured in the presence of ADR at a concentration of 3 ng/ml for 0, 1, 3, 6, and 12 h. Podocytes were lysed on ice in lysis buffer, and then protein samples were subjected to SDS-PAGE and WB analysis. β-Actin was measured in cell lysates that were used as controls.

### Mouse model of ADR-induced proteinuria

ADR (doxorubicin hydrochloride; Wako, Osaka, Japan) was diluted in 0.9% saline and was administrated to non-anesthetised wild-type and Rac1 conditional-knockout (cKO) mice without detectable glomerulosclerosis at a dose of 8–9 mg/kg body weight (BW) via tail-vein injection. Rac1^flox/flox^ mice were used as the control. Urine was collected once every few days. The urinary albumin/creatinine ratio (ACR) was measured using an immunoassay (DCA 2000 Systems; Siemens Medical Solutions Diagnostics, Tarrytown, N.Y., USA) with a Bayer DCA 2000+ chemical analyser (Bayer Diagnostics, Elkhart, Ind., USA). After anesthesia with sodium pentobarbital (100 mg/kg BW; Dainippon Sumitomo Pharma, Osaka, Japan), 3–5 mice per day were euthanised on days 0, 7, 14, 21, and 28 after ADR injection. All mice were housed under specific pathogen-free conditions using standard animal cages with free access to standard chow and drinking water.

### Renal histology and immunohistochemistry

Kidneys fixed in 4% paraformaldehyde and 20% sucrose were paraffin-embedded and sectioned at a thickness of 3 μm. The sections were stained using a periodic acid-Schiff (PAS) method and were observed by light microscopy (LM) (BX41 microscope; Olympus, Tokyo, Japan). To determine the frequency of glomerulosclerosis, the number of glomeruli with sclerosis per total number of glomeruli was determined in 4 mice. The extent of adhesion of a capillary to Bowman’s capsule was determined as the number of adhesions per total number of glomeruli in 3 mice.

For immunofluorescence (IF) experiments, the kidneys were fixed in the same reagent as that used for LM, placed in Optimum Cutting Temperature compound (Sakura Fintec Japan, Tokyo, Japan), frozen in liquid nitrogen, and stored at −80 °C. Double IF staining of Rac1, podocyte-associated proteins, and mTOR-pathway proteins, as well as post-staining with DAPI (4′,6-diamidine-2-phenylindole) were carried out as described previously^26^. The sections at a thickness of 3 μm were observed under a confocal laser microscope (Olympus FV1000; Olympus). To examine the number of podocytes in glomeruli, cells double-positive for Wilm’s tumor 1 (WT-1) and DAPI staining were counted in over 100 glomeruli obtained from 3–6 mice. To examine the ratio of phosphorylated (p) S6 positive glomeruli, pS6 and synaptopodin double-positive glomeruli among total glomeruli were determined. Nuclei were stained with DAPI. The glomerular tuft area and synaptopodin positive area were measured using KS400 image analysis software (Zeiss). Glomerular volume, glomerular volume per podocyte (GV/P), and individual podocyte volume were measured as previously described^20,27^. In the present study, synaptopodin antibody was used to define podocytes.

For electron microscopy (EM), after perfusion fixation with 4% paraformaldehyde, small blocks of kidney tissue were fixed with 2% glutaraldehyde and postfixed in 1% OsO_4_. The samples were dehydrated in a graded ethanol series and embedded in epoxy resin. Ultrathin sections (80–100 nm thick) were stained with uranyl acetate and lead citrate, and were examined by EM (H-7700; Hitachi, Tokyo, Japan) at 100 kV. At least 3–4 glomeruli per sample were analysed, and the extent of detachment was assessed quantitatively by measuring the length of detachment area per GBM in 3 mice. The length of the entire GBM and the areas of detachment were measured using Image-J software (Rasband, W.S., ImageJ, U. S. National Institutes of Health, Bethesda, MD, USA).

### WB analysis

To detect Rac1 expression, glomeruli were isolated from kidneys using magnetic beads and lysed^28^. Rac1 and GAPDH expression were assessed by WB analysis as described previously^29^, with GAPDH expression serving as an internal control.

### Rac1 activation assay

Rac1 activity was evaluated using a Rac1 activation assay. Differentiated podocytes were treated with 3 ng/ml of ADR for 6 h to induce cell damage. Rac1 activity of cultured podocytes in the presence or absence of ADR was assessed using a Rac1 activation assay kit (Cytoskeleton Inc., Denver, CO). Cell lysate (500 mg protein) was prepared and mixed with PAK1-PBD-immobilised beads according to the manufacturer’s protocol. The mixture was incubated at 4 °C for 1 h and centrifuged at 15,000 × *g* for 3 min. The beads were washed once with washing buffer and centrifuged again. The beads were then treated with SDS-PAGE sample buffer and solubilised protein was subjected to WB analysis for bead-bound (activated) Rac1.

### Statistical analysis

All data are expressed as the mean ± SEM. Statistical significance (defined as *p* < 0.05) was evaluated using Student’s t-test.

## Results

### Podocyte-specific ablation of Rac1 does not cause congenital nephropathy

**Figure 1:**
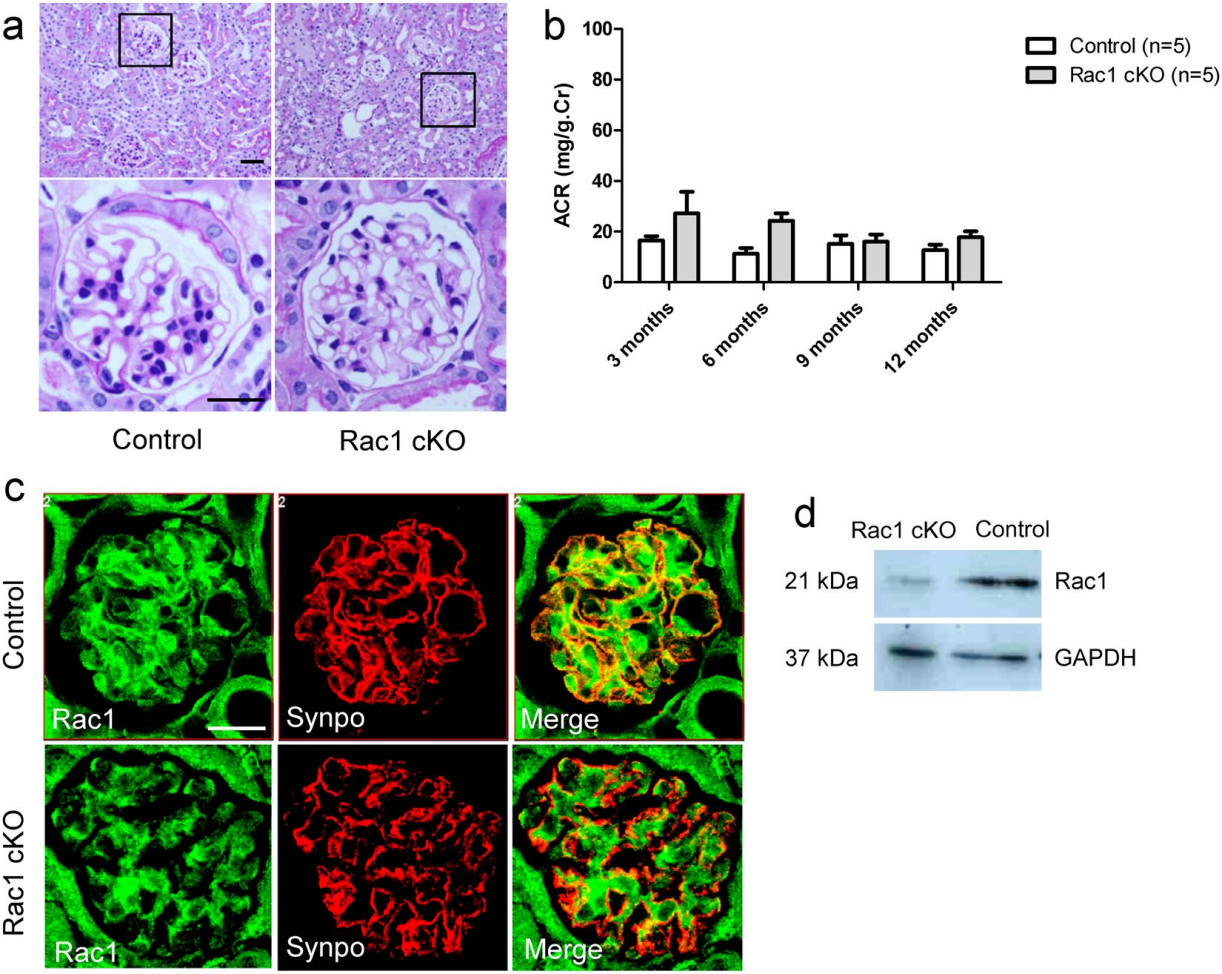
Comparison of Rac1 cKO mice and control littermates under physiological conditions. (**a**) PAS staining of kidney sections showed similar morphologly of Rac1 cKO mice and control littermates. Scale bar, 50 μm (upper panel), 20 μm (lower panel). (**b**) Rac1 cKO mice had urinary protein levels similar to those in control mice (*p* > 0.05, n = 5). (**c**) IF staining of Rac1 and synaptopodin in Rac1 cKO mice and control mice. Upper panels: in control mice, Rac1 co-localised with synaptopodin. Lower panels: in Rac1 cKO mice, Rac1 did not co-localise with synaptopodin. Scale bar, 20 μm. (**d**) WB analysis demonstrated that Rac1 expression was lower in Rac1 cKO mice than in control mice.

Nphs2-Cre: Rac1^flox/flox^ cKO mice were born at an expected Mendelian ratio and were largely indistinguishable from control littermates. Compared with control mice, kidney sections from Rac1 cKO mice showed no differences in glomeruli and tubules under basal conditions as indicated by PAS staining (Fig. 1a). Moreover, Rac1 cKO mice showed no overt deterioration in health relative to control mice and did not develop proteinuria, defined as urinary ACR by 12 months of age (*p* = 0.34, 0.40, 0.94, and 0.67 at 3, 6, 9, and 12 months, respectively, n = 5 at each time point, Fig. 1b). IF experiments revealed that Rac1 and synaptopodin colocalised at the glomeruli in control mice, but not in Rac1 cKO mice (Fig. 1c). WB analysis demonstrated that Rac1 expression was lower in Rac1 cKO mice than in control mice (Fig. 1d).

### Injection of ADR causes a loss of podocytes from the GBM and glomerulosclerosis in Rac1 cKO mice

**Figure 2:**
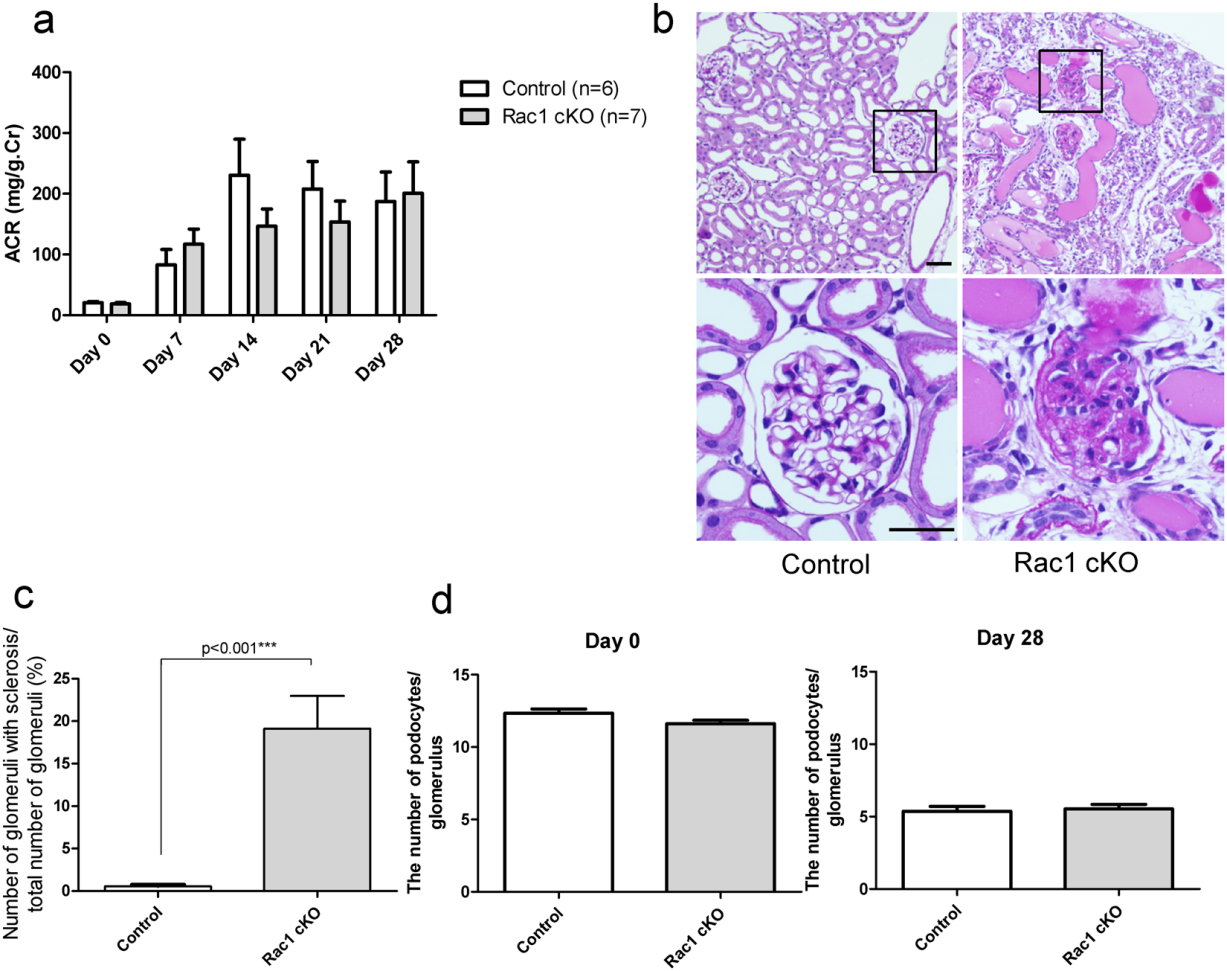
Phenotypic alterations of Rac1 cKO and control mice after ADR administration. (**a**) Urinary protein levels gradually increased, but were not significantly different between Rac1 cKO mice and control mice. (**b**) Left panels: histological examination of kidney tissues from Rac1 cKO mice on day 28 post-ADR administration revealed some sclerotic glomeruli. Right panels: conversely, few sclerotic glomeruli were observed in control mice on day 28 post-ADR administration. Scale bar, 50 μm (upper panel), 20 μm (lower panel). (**c**) The percentage of sclerotic glomeruli was higher in Rac1 cKO mice than in control mice on day 28. (**d**) The number of podocytes per glomerulus was not significantly different between Rac1 cKO mice and control mice on either day 0 or day 28.

**Figure 3:**
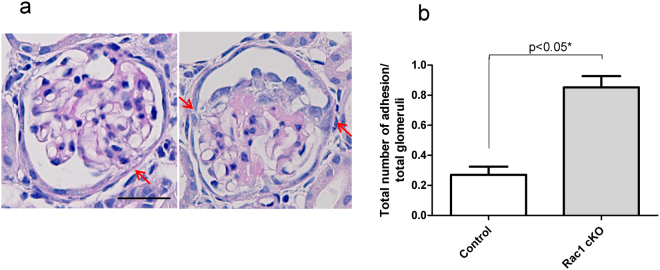
More adhesion of the GBM to Bowman’s capsule was observed in Rac1 cKO than in control mice on day 14 post-ADR administration. (**a**) Periodic acid methenamine silver staining of the glomeruli in Rac1 cKO mice showed some adhesion of the GBM to Bowman’s capsule. Adhesion at the 4 o’clock position (red arrow, left panel). Adhesion at the 3 o’clock and 9 o’clock positions (red arrows, right panel). Scale bar, 20 μm. (**b**) More adhesion of the GBM to Bowman’s capsule was observed in Rac1 cKO mice than in control mice on day 14.

We next investigated the podocyte injury phenotype in Rac1 cKO mice. We administered ADR, which causes podocyte loss from the GBM and glomerulosclerosis, to both Rac1 cKO mice and control mice. The urinary protein levels increased gradually and were not significantly different between Rac1 cKO and control mice (*p* = 0.57, 0.32, 0.15, 0.17, and 0.57 at days 0, 7, 14, 21, and 28, respectively, n = 7 Rac1 cKO mice, n = 6 control mice; Fig. 2a). Histologic alteration of the kidneys in Rac1 cKO mice on day 28 after ADR administration indicated some sclerotic glomeruli. Conversely, few sclerotic glomeruli were observed in control mice on day 28 (Fig. 2b). The ratio of glomerulosclerosis was significantly higher in Rac1 cKO than in control mice (0.56 ± 0.23% in control mice versus 19.12 ± 3.85% in Rac1cKO mice, *p* < 0.001; Fig. 2c). Next, we counted the remaining podocytes per glomerulus to evaluate the loss of podocytes after podocyte injury. The number of podocytes per glomerulus was not significantly different between Rac1 cKO and control mice on day 0 (12.34 ± 0.29 in control mice versus 11.62 ± 0.24 in Rac1cKO mice, *p* = 0.08; Fig. 2d) or on day 28 (5.37 ± 0.34 in control mice versus 5.53 ± 0.31 in Rac1cKO mice, *p* = 0.92; Fig. 2d). It is well known that adhesion of the GBM to Bowman’s capsule leads to glomerulosclerosis^3^. To explore the process of sclerosis formation, we quantified the extent of adhesion. Some adhesions were observed in Rac1 cKO mice on day 14 (arrows, Fig. 3a). On day 14, significantly more adhesions were observed in Rac1 cKO than in control mice (0.27 ± 0.05 in control versus 0.85 ± 0.07 in Rac1 cKO; *p* < 0.05; Fig. 3b).

### Podocyte morphology in Rac1 cKO mice and control mice administered ADR

**Figure 4:**
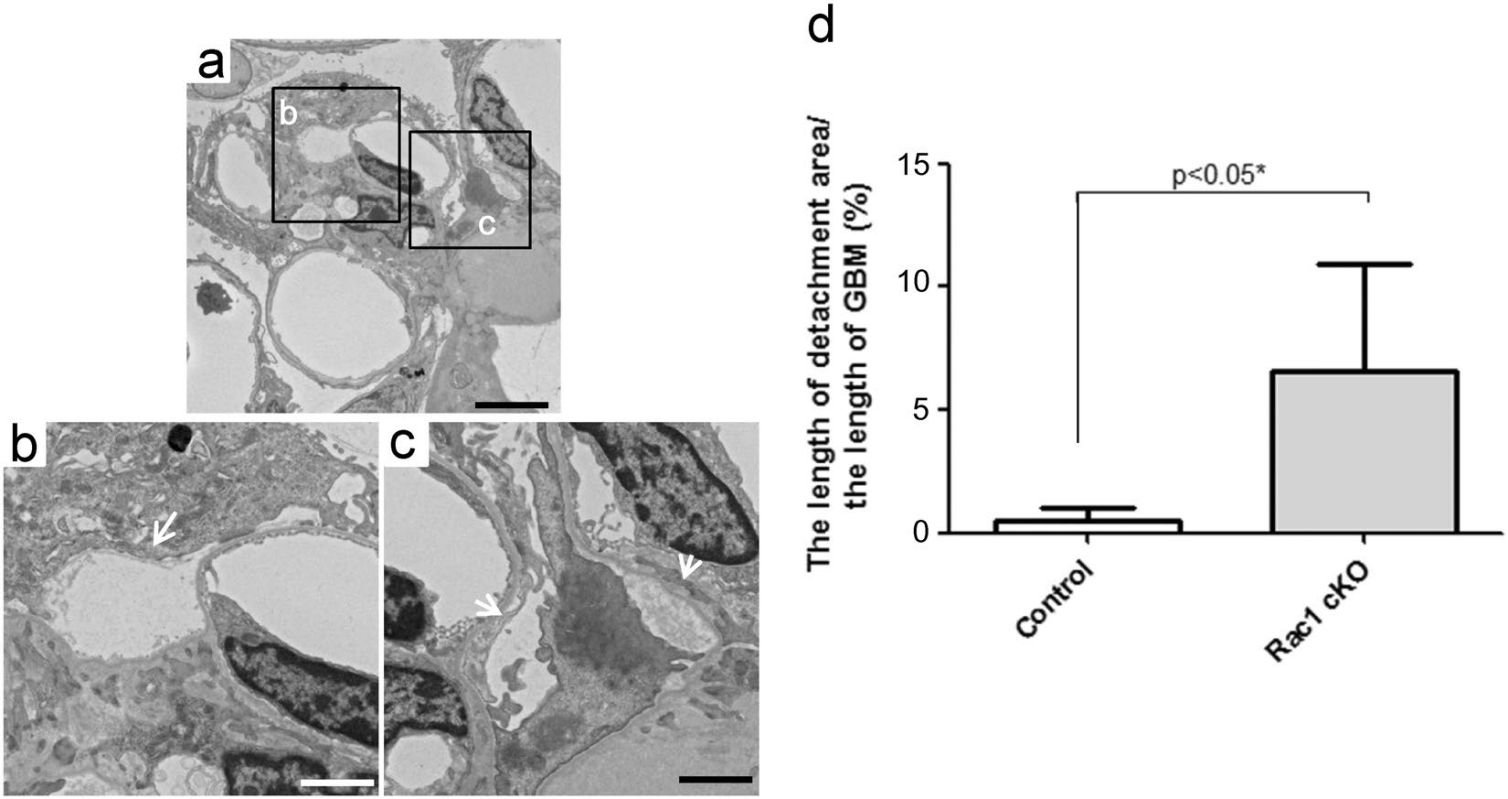
EM results showed more podocyte detachment from the GBM in Rac1 cKO mice than in control mice on day 28. (**a**) Podocyte detachment areas are detected in Rac1 cKO mice on day 28. Scale bars: 5 μm. (**b**,**c**) Magnified views of the detached areas marked with arrows in (**a**). Scale bars: 2 μm. (**d**) Detachment area was larger in Rac1 cKO mice than in control mice.

Morphological alterations of podocytes in Rac1 cKO and control mice that were administered ADR, were examined by EM. Podocyte detachment from the GBM is the first step in glomerulosclerosis development^3^. Thus, we compared the ratio of podocyte detachment in Rac1 cKO and in control mice. Some podocyte detachment areas were observed in Rac1 cKO mice on day 28 (Fig. 4a,b,c). The ratio of detachment in Rac1cKO mice exceeded that in control mice [6.6 ± 4.3% in Rac1cKO mice (n = 3) versus 0.5 ± 0.5% in control mice (n = 3); *p* < 0.05, Fig. 4d].

### S6 phosphorylation and podocyte volume were reduced in Rac1 cKO mice in response to ADR administration

**Figure 5:**
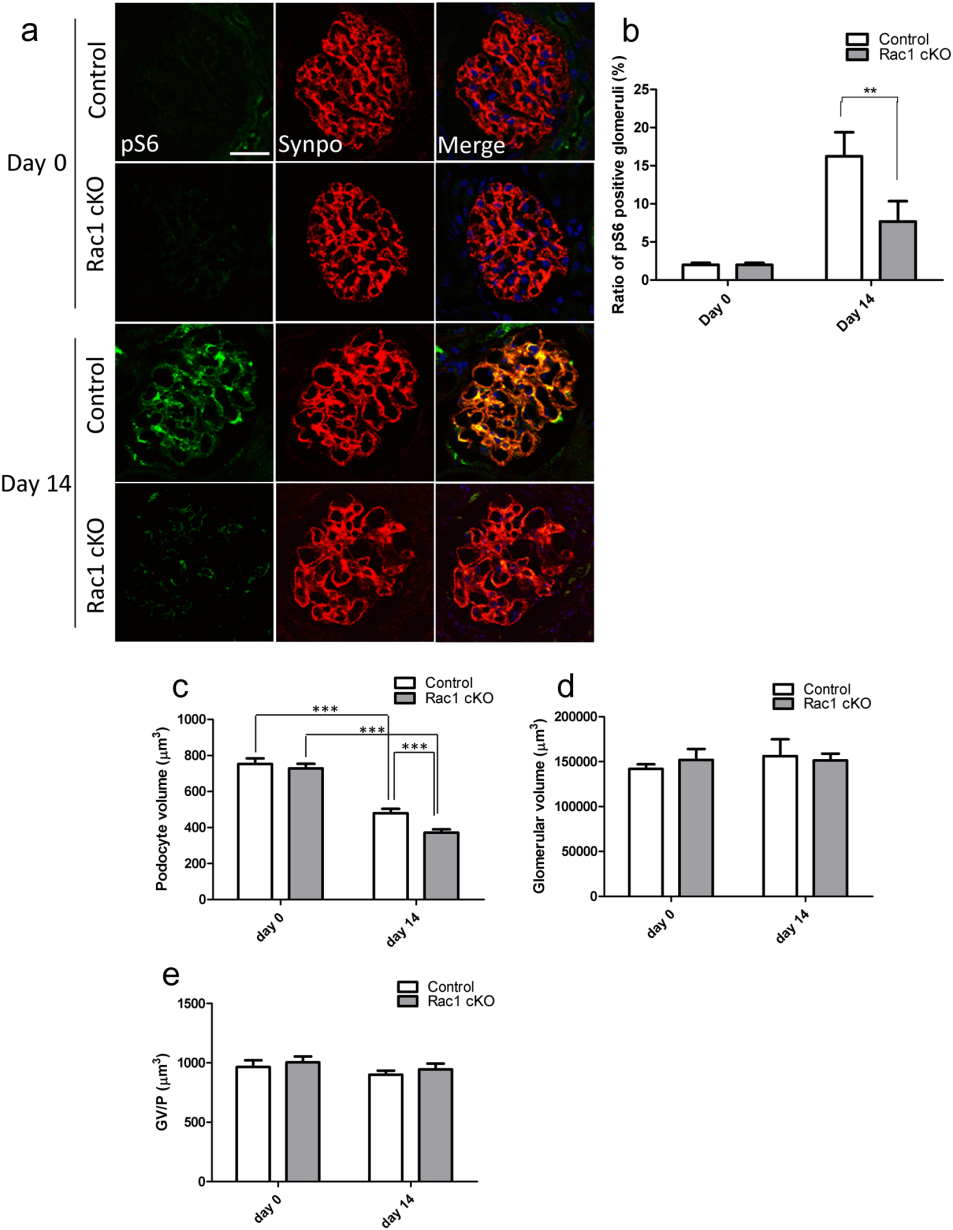
S6 phosphorylation after ADR administration is lower in Rac1 cKO mice than in control mice. (**a**) pS6 (green), synaptopodin (Synpo; red), and DAPI (blue) staining of kidney sections of mice with ADR induced nephropathy at the indicated time points. On day 14, pS6 was detected readily in control mice, while little pS6 was detected in Rac1 cKO mice. Scale bar, 20 μm. (**b**) The ratio of pS6 positive glomeruli was lower in Rac1 cKO mice than in control mice on day 14 post-ADR treatment (n = 6 per group, *p* < 0.01). (**c**) The individual podocyte volume was reduced in control mice and Rac1 cKO mice on day 14 (n = 3 per group, *p* < 0.0001 in control and Rac1 cKO). The individual podocyte volume was greater in control mice than in Rac1 cKO mice on day 14 (n = 3 per group, *p* = 0.0001). (**d**) Glomerular tuft volume did not significantly differ between control mice and Rac1 cKO mice on day 0 or day 14 (n = 3 per group). (**e**) Glomerular tuft volume/podocyte (GV/P) did not significantly differ between control mice and Rac1 cKO mice on day 0 or day 14 (n = 3 per group). **p < 0.01, ***p < 0.001.

Cell size is regulated by the mTOR pathway through mTORC1-dependent phosphorylation of S6 and the eukaryotic translation initiation factor 4E-binding protein (4E-BP), which together stimulate ribosome biogenesis and protein translation to increase cell mass^19^. Gödel *et al*. reported that pS6 can serve as a marker of mTORC1 activity and confirmed increased pS6 levels in podocytes from patients and animal models with diabetic nephropathy^30^. We hypothesized that the remaining podocytes increase their size under pathological condition to compensate the glomerular dysfunction due to denuded GBM. To investigate whether the remaining podocytes change their size, we immunostained glomeruli in control and Rac1 cKO mice administered ADR for pS6. IF staining showed that pS6 colocalized with synaptopodin on day 14 post-ADR administration, especially in control mice (Fig. 5a). The fraction of pS6-positive glomeruli in control mice was higher than that in Rac1 cKO mice on day 14 [16.22 ± 3.17% in control mice (n = 6) versus 7.67 ± 2.67% in Rac1 cKO mice (n = 6), *p* < 0.01, Fig. 5b]. These results indicated that Rac1 deletion suppressed S6 phosphorylation.

Since S6 phosphorylation is a marker of growth in cell size, we estimated the volumes of individual podocytes in control mice and Rac1 cKO mice on day 0 and day 14 post-ADR administration. There was no significant difference in the volumes of individual podocytes between control mice (n = 3) and Rac1 cKO mice (n = 3) on day 0 (*p* = 0.53, Fig. 5c). Upon ADR administration, podocyte volume was reduced on day 14 in both control mice (n = 3) and Rac1 cKO mice (n = 3 per group, *p* < 0.0001 for both, Fig. 5c). Nevertheless, podocyte volume was still significantly larger in control mice than in Rac1 cKO mice (*p* = 0.0001, Fig. 5c). To evaluate glomerular tuft volume changes after ADR administration, we measured glomerular tuft volume and glomerular tuft volume per podocyte (GV/P). Glomerular tuft volume did not differ between control mice and Rac1 cKO mice and did not differ between day 0 and day 14 (*p* = 0.51, control vs. cKO on day 0; *p* = 0.80, control vs. cKO, on day 14; *p* = 0.08, day 0 vs. day 14 in control mice; *p* = 0.86, day 0 vs. day 14 in cKO mice Fig. 5d). GV/P was also unaltered in control mice and Rac1 cKO mice from day 0 to day 14 (*p* = 0.43, control vs. cKO on day 0; *p* = 0.45, control vs. cKO on day 14; *p* = 0.37, day 0 vs. day 14 in control mice; *p* = 0.70 day 0 vs. day 14 in cKO mice, Fig. 5e). These results indicate that Rac1 in podocytes contributes to preventing ADR-induced reduction of podocyte volume in mTORC1-dependent manner.

### Rac1 KD suppresses S6 phosphorylation in cultured podocytes treated with ADR

**Figure 6:**
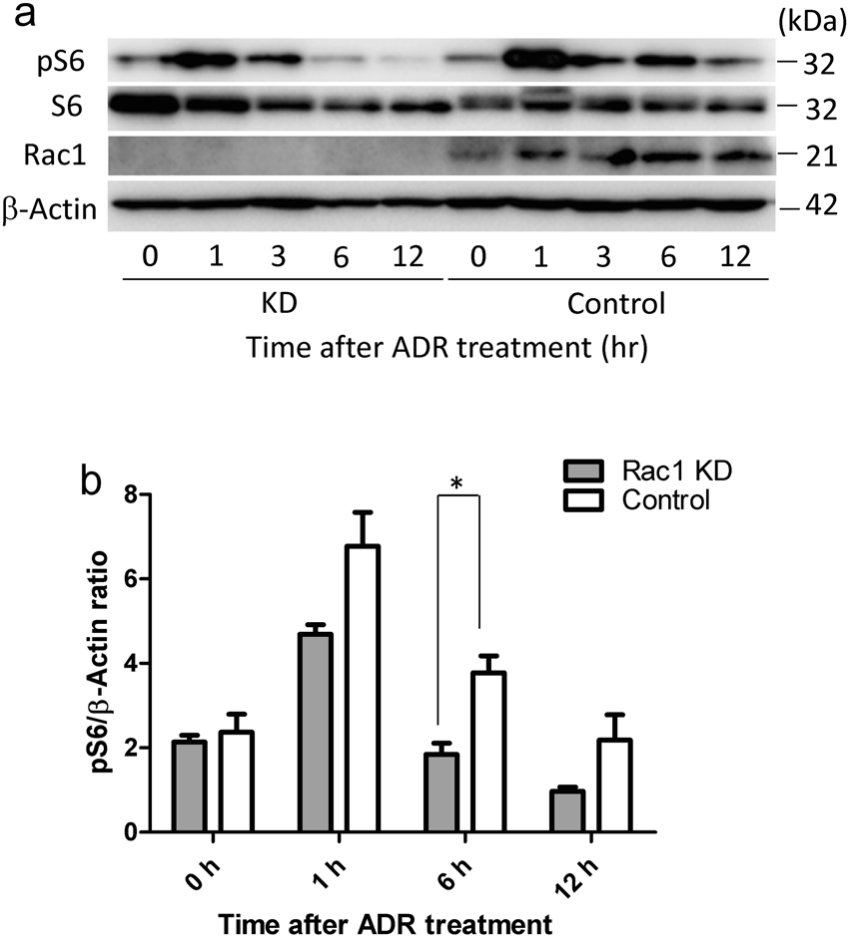
Rac1 KD suppressed S6 phosphorylation in response to ADR. (**a**) pS6 levels increased at 1–3 h and decreased at 6 h after ADR treatment in Rac1 KD podocytes. In contrast, pS6 levels increased at 1–6 h after ADR treatment in control podocytes. (**b**) Quantitative data for pS6/β–actin ratio are shown. pS6 levels in Rac1 KD podocytes were lower than that in control podocytes at 6 h after ADR treatment (p < 0.05). There is no significant difference in pS6 level between Rac1 KD and control podocytes at 0 h (*p* = 0.49), 1 h (*p* = 0.08), 12 h (*p* = 0.22). Data are presented as the mean ± SEM (n = 3). *p < 0.05.

To elucidate how Rac1 affects the mTOR system in injured podocytes, we next investigated this issue using cultured podocytes. We produced Rac1 KD podocytes using a shRNA-expressing adenovirus vector. Rac1 KD and control podocytes were treated with 3 ng/ml ADR to induce cell damage. WB analysis showed that pS6 increased at 1–3 h after ADR treatment in Rac1 KD podocytes and at 1–6 h in control podocytes (Fig. 6a). pS6/β–actin ratio was lower in Rac1 KD than in control podocytes with statistical significance at 6 h after ADR treatment (n = 3 per group, *p* < 0.05, Fig. 6b). These results suggested that podocyte damage induced by ADR triggers mTORC1 pathway activation but that the activation was diminished in Rac1 KD podocytes.

### Rac1-GTP expression is not elevated at 6 h after ADR treatment

**Figure 7:**
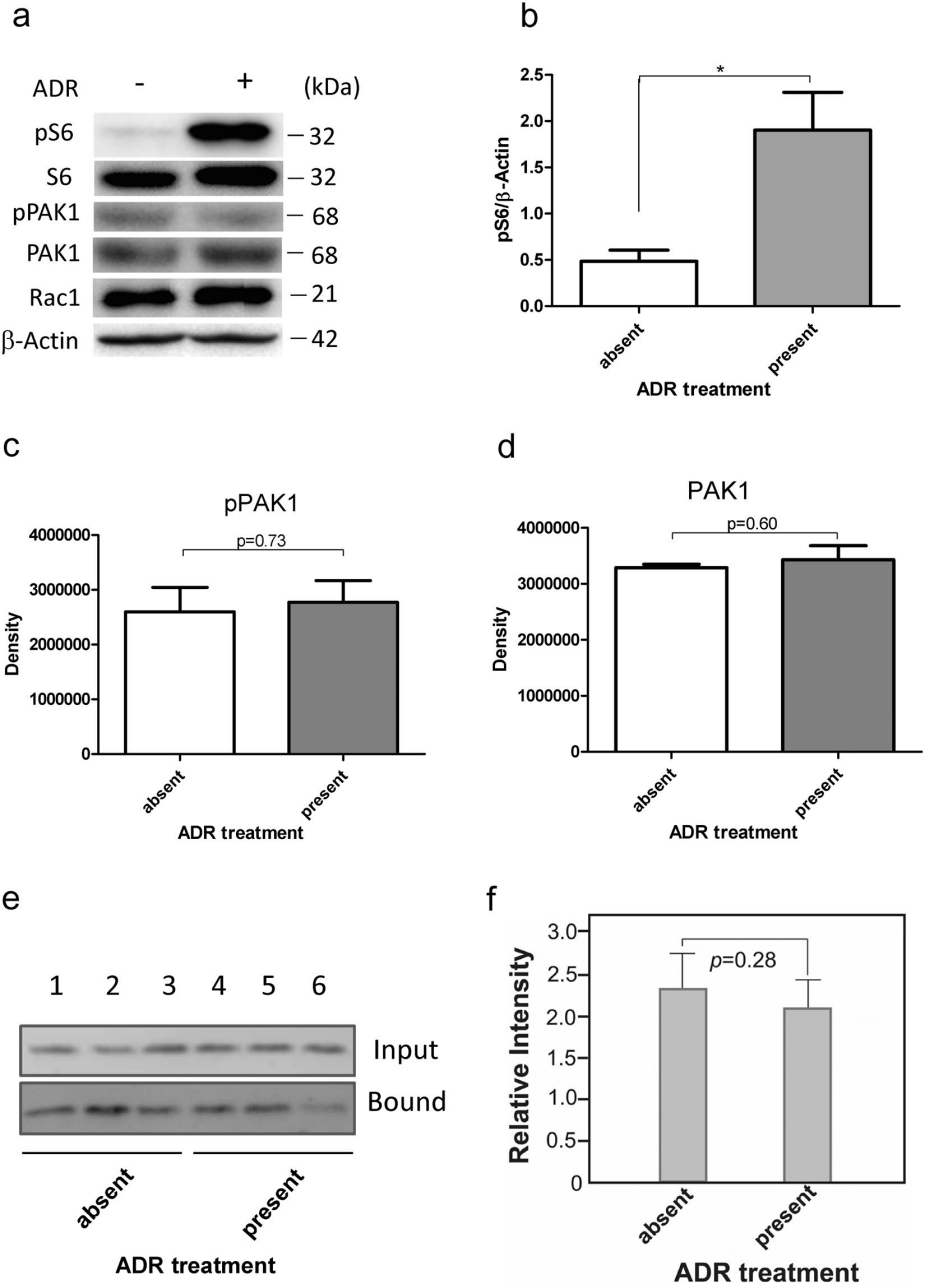
Rac1-GTP expression was not elevated at 6 h after ADR treatment. (**a**) S6 phosphorylation was induced at 6 h after ADR treatment, while phosphorylated PAK1 was barely observed at this time point. (**b**) Quantitative densitometry analysis of pS6/β–actin ratio is shown (p < 0.05). Data are presented as the mean ± SEM (n = 4). *p < 0.05. (**c**) Quantitative densitometry analysis of pPAK1 is shown (*p* = 0.73). Data are presented as the mean ± SEM (n = 3). (**d**) Quantitative densitometry analysis of PAK1 is shown (*p* = 0.60). Data are presented as the mean ± SEM (n = 3). (**e**) Podocyte lysates were prepared from 3 independent cultures in the absence^1–3^ or presence^4–6^ of ADR. Lysates (8 mg protein) were analysed for total Rac1 by WB analysis (Input). The same lysates (500 mg protein) were incubated with PAK1-PBD-immobilised beads, centrifuged, and washed as described in Materials and Methods. Bead-bound Rac1 (activated Rac1, Bound) was analysed by WB analysis. (**f**) Densitometric data for activated Rac1 precipitated with PAK1-PBD beads (n = 3).

Rac1 exists in two interconvertible forms: a GDP-bound inactive form and a GTP-bound active form. As described above, we showed that pS6 levels were significantly decreased in Rac1 KD compared with control podocytes at 6 h after ADR treatment (Fig. 6b). In addition, in control podocyte, pS6 is increased at 6 h after ADR treatment (*p* < 0.05, Fig. 7b). It is therefore interesting to investigate, whether or not Rac1 activation is necessary for S6 phosphorylation ( = mTOR activation) in control ADR-treated podocytes. PAK1, which is a major downstream protein of the Rho GTPases Cdc42 and Rac1, is activated by binding to GTP-bound Rac1 to become phosphorylated. As shown in Fig. 7a, pPAK1 and PAK1 levels were not changed at 6 h after ADR treatment (n = 4 per group, *p* = 0.73, Fig. 7c; n = 4 per group, *p* = 0.06, Fig. 7d). Next, we performed an activated Rac1 pull-down assay using PAK1-PBD-immobilized beads, which specifically binds active Rac1-GTP form. Data obtained at 6 h after treatment with or without ADR (Fig. 7d) clearly show that Rac1-GTP was not significantly increased by ADR treatment (n = 3 per group, *p* = 0.28, Fig. 7e).

## Discussion

The Rac1 pathway has been studied intensely and has significant relevance to human glomerular diseases. Examples include studies of the aldosterone receptor pathway in podocytes^17^, as well as studies of the urokinase plasminogen activator receptor^31,32^. Rho GDP dissociation inhibitor (RhoGDI) interacts with GDP-bound inactivated Rho GTPases and prevents their conversion to the active GTP-bound form. RhoGDI-α deficient mice, which show increased Rac1 activation in the kidneys, were found to develop proteinuria, foot process (FP) effacement, and glomerulosclerosis indicating severe podocyte damage^17,33^. KD of RhoA-activated Rac1 GTPase-activating protein 24 (Arhgap24) increased the levels of active Rac1 and Cdc42 in podocytes. Arhgap24 is up-regulated in differentiated podocytes. An inactivating mutation of the ARHGAP24 gene was detected in a family with focal segmental glomerular sclerosis^34^. Podocyte FP plasticity is recognised as an important feature in regulating FP dynamics and filter function^35,36^. Podocyte-specific Akt2 deletion accelerated podocyte injury, including apoptosis and FP effacement^37^. Yu *et al*. reported that podocyte-specific constitutively active Rac1 expression in mice caused a rapid onset of proteinuria with FP effacement, but the proteinuria was transient^38^. Loss of podocyte-specific Rac1 protects against induction of podocyte FP effacement by protamine sulfate^14^. These results suggested that Rac1 pathways play important roles in the early development of proteinuria and FP effacement. Given the necessary balance of Rho GTPases in podocytes, it is worth considering whether the deletion of Rac1, even during early stages with no injury, is compatible with long-term renal health and with the ability to undergo repair following injury. In a model of long-term chronic hypertensive glomerular damage, loss of Rac1 led to exacerbated of albuminuria and glomerulosclerosis^14^.

In the present study, ADR administration to Rac1 cKO mice significantly increased the number of sclerotic glomeruli. In a rat model of tunable podocyte loss, approximately 20–40% of podocyte deletion was required before focal segmental glomerular sclerosis could be consistently observed^37^. In the present study, the scale of podocyte deletion was similar in ADR-injected Rac1 KO and control mice. However, the number of sclerotic glomeruli was significantly higher in Rac1 cKO mice than in control mice (Fig. 2c). These results suggested that the presence of Rac1 in podocytes protected injured glomeruli against the progression of glomerulosclerosis under pathological conditions. These results raise the question of how the presence of Rac1 in podocytes prevents glomerulosclerosis progression in ADR-induced nephrosis. Larger areas of detached podocytes (Fig. 4) and more adhesion of the GBM to Bowman’s capsule (Fig. 3) were observed in Rac1 cKO mice than in control mice after ADR treatment. However, there was no significant difference in the number of remaining podocytes after ADR treatment between the two groups (Fig. 2d,e). As podocytes are terminally differentiated cells, we speculated that the only way to compensate for a denuded GBM, or detached podocyte area, would be expansion or hypertrophy of individual podocytes, which cover denuded GBM area.

Cell size is regulated by the mTOR pathway through mTORC1-dependent S6K phosphorylation and 4E-BP, which stimulate ribosome biogenesis and protein translation to increase cell mass^39^. Inoki *et al*. reported that the genetic reduction of podocyte-specific mTORC1 in diabetic animals suppressed the development of diabetic nephropathy *in vivo*^40^. Gödel *et al*. reported that deletion of genes in mTORC1 induced progressive glomerulosclerosis in mouse podocytes, whereas curtailing mTORC1 signaling by genetically reducing the mTORC1 copy number in mouse podocytes prevented glomerulosclerosis and significantly ameliorated the progression of glomerular diseases in diabetic nephropathy^30^.

Our data revealed that S6 phosphorylation, as a marker of mTORC1 activity, was induced in damaged control mice in the presence of Rac1, whereas S6 phosphorylation was markedly diminished in Rac1 cKO mice (Fig. 5a,b). These results were further supported by podocyte volume measurements (Fig. 5c). Although ADR induced reduction in podocyte volume in both control and Rac1 cKO mice, scale of reduction is significantly larger in Rac1 cKO mice than in control mice (Fig. 5c). The data strongly suggest that Rac1 in podocytes contributes to preventing ADR-induced atrophy, probably through activation of mTOR pathway. Lack of mTOR activation in Rac1 cKO podocytes is consistent with a previous study reporting that Rac1 deletion inhibits the activation of downstream targets of mTOR in cultured fibroblasts and lymphocytes^19^.

Whether or not Rac1 activity is required for mTOR activation in podocytes is important. We attempted to investigate this issue using cultured podocytes. Basically, during 12 hr period of post ADR administration, phosphorylated S6 was more increased in control podocytes than in Rac1 KD podocytes (Fig. 6a,b), being consistent with *in vivo* data. Next, we compared control podocytes with or without ADR administration. However, no obvious activation of Rac1 was recognized before and after 6 hr ADR treatment (Fig. 7). Although we cannot rule out the possibility that Rac1 is activated at earlier periods of ADR treatment precedential to S6 phosphorylation, our data may indicate that the presence of Rac1, rather than Rac1 activity, is necessary for mTOR activation as described in a previously study by Saci *et al*. in which Rac1 binds directly to mTOR to control cell size and that the binding of Rac1 to mTOR does not depend on the GTP-bound state of Rac1^19^.

Fukuda *et al*. reported that insufficient podocyte hypertrophy in relation to glomerular tuft growth could lead to glomerulosclerosis using a uninephrectomy model^20^. However, we found that podocyte volume was altered while glomerular tuft volume was not altered after ADR treatment. One possible explanation for this discrepancy may be the different type of disease models used.

A limitation of our study is that we did not examine the actin cytoskeleton in injured podocytes. Rac1 is known to regulate actin organization; therefore, further studies should investigate the role of Rac1 in injured podocytes with regard to the actin cytoskeleton.

In summary, our data demonstrate that, in injured podocytes, the presence of Rac1 promotes mTOR activation so that remaining podocytes could maintain their size to protect the kidneys from developing glomerulosclerosis. Rac1 and mTOR require each other to maintain homeostasis in podocytes and exhibit potential as therapeutic targets for glomerulosclerosis.
